# Immune characteristics correlating with HSV‐1 immune control and effect of squaric acid dibutyl ester on immune characteristics of subjects with frequent herpes labialis episodes

**DOI:** 10.1002/iid3.241

**Published:** 2019-02-12

**Authors:** Hugh McTavish, Katherine W. Zerebiec, Jay C. Zeller, Laurie L. Shekels, Mark A. Matson, Betsy T. Kren

**Affiliations:** ^1^ Squarex, LLC Saint Paul Minnesota; ^2^ Center for Veterans Education and Research Veterans Administration Medical Center Minneapolis Minnesota; ^3^ Prism Research St. Paul Minnesota; ^4^ Minneapolis Veterans Affairs Health Care System Minneapolis Minnesota; ^5^ Masonic Cancer Center University of Minnesota Minneapolis Minnesota

**Keywords:** squaric acid dibutyl ester, herpesvirus 1, herpes labialis, herpes simplex, interferon gamma, interleukin‐5, squaric acid

## Abstract

**Introduction:**

Differences in immune characteristics, including immune gene expression by peripheral blood mononuclear cells (PBMCs), correlating with herpes labialis and good or poor immune control of herpes simplex virus type 1 (HSV‐1), and how these characteristics change after dosing with squaric acid dibutyl ester (SADBE), were investigated.

**Methods:**

PBMCs were collected from persons positive for IgG against HSV‐1 and having frequent, infrequent, or no herpes labialis outbreaks. The PBMCs were tested for proliferation against HSV‐1 and a fungal antigen (*Candida*) and immune gene expression in the presence of HSV‐1 and *Candida*. On day 1 after blood collection the subjects with frequent outbreaks were dosed topically on the arm once with SADBE, and their PBMCs were collected and tested 8 weeks later.

**Results:**

Those with good immune control of their HSV‐1 infection (fewer outbreaks) differ from those with poorer immune control in these ways: (1) Greater PBMC proliferation in vitro to HSV‐1, HSV‐1‐infected cell extracts, and *Candida* considered together (*P* < 0.01). (2) Higher expression of IFNG and five other immune‐related genes (*P* < 0.05 for each) and lower expression of IL5 and two other immune‐related genes (*P* < 0.05 for each) in PBMCs in vitro stimulated with HSV‐1 virus. The subjects with frequent outbreaks were treated once with SADBE, and 56 days later the PBMCs of these subjects differed from PBMCs from the same subjects taken on day 1 before treatment in exactly the same ways listed above as differences between those with good and poor immune control of HSV‐1, and at the same levels of significance.

**Conclusions:**

Higher IFNG and lower IL5 expression by PBMCs in the presence of HSV‐1 correlate with fewer herpes labialis outbreaks, and a single topical dose of SADBE to the arm of subjects with frequent herpes labialis episodes improves immune response to HSV‐1.

## 1 INTRODUCTION

Herpes labialis is a common condition characterized by blisters or erosions on the lips and skin around the mouth and nose.^1^, ^2^, ^3^ Most cases are caused by herpes simplex virus type 1 (HSV‐1), but 10–15% of cases are caused by HSV‐2 with this percentage reportedly increasing.^2^ In a population survey in the U.S., 76% of adults were positive for IgG antibodies against HSV‐1.^4^ A population survey of over 10 000 randomly selected adults in France found 14.8% had a herpes labialis episode in the previous 12 months, and of those with herpes labialis, 12.94% had 6 or more occurrences in the last 12 months.^5^

The natural history of HSV‐1 infection leading to herpes labialis is that the virus typically initially infects oral mucosa tissue, and then migrates to sensory neurons and establishes latency in sensory neurons, typically the trigeminal ganglion. The virus is later activated from latency by events including fever, stress, cold or flu infection, immunosuppression, and sunlight. Upon activation, the virus migrates down sensory neurons to epithelial cells, typically on the vermillion border of the lip, and causes cell lysis and outbreaks as epidermal lesions there.^6^ Outbreaks typically last 1–2 weeks. The frequency and severity of outbreaks is thought to be dependent on the effectiveness of immune control of the virus.^7^, ^8^ McKenna showed that persons with frequent outbreaks had lower lymphoproliferation in vitro in the presence of inactivated HSV‐1 and lower interferon‐gamma protein levels in vitro in the presence of inactivated HSV‐1 than persons with infrequent outbreaks.^7^

In a previous placebo‐controlled clinical trial, topical application of a 2% solution of squaric acid dibutyl ester (SADBE) in dimethylsulfoxide (DMSO), applied once to the arm of persons who self‐reported 6 or more herpes labialis outbreaks over the previous 12 months, was found to significantly delay the next outbreak. The median time to the next outbreak for the placebo group was 40 days vs more than 122 days for the 2.0% SADBE group, which difference was highly significant (*P* = .009).^9^ SADBE is a topical immunosensitizer that induces a delayed‐type hypersensitivity response and is commonly used in the treatment of verruca vulgaris and alopecia areata.^10^, ^11^, ^12^

In this study, we first sought to determine differences in immune function in general, and in immune response to HSV‐1 in particular, between persons who are infected with HSV‐1 and have frequent herpes labialis episodes (6 or more outbreaks in previous 12 months, group A) as compared to persons who are infected with HSV‐1 and have infrequent herpes labialis episodes (1 or 2 outbreaks in previous 12 months, group B) or infected with HSV‐1 and have no herpes labialis episodes (0 outbreaks in previous 12 months, group C). Second, we also sought to determine the effects on the immune system in general and on immune response to HSV‐1 virus in particular of a single topical application of 2% SADBE in DMSO to the arm.

The outcomes measured were:

Blood levels:

- Blood cell counts, including lymphocytes, T cells, B cells, CD4, CD8, and natural killer cells.
- Serum anti‐HSV‐1 IgG quantitative levels.
- Plasma cytokine levels.

Peripheral blood mononuclear cell (PBMC) proliferation in vitro in response to:

- HSV‐1‐infected cell extracts (heat inactivated)
- HSV‐1 virus, cell‐free (heat inactivated)
- *Candida albicans* extract (a common infectious organism whose extract is used to measure general immune function).

Cytokine and immune‐function gene expression of PBMC in vitro in response to:

- Medium only negative control
- HSV‐1‐infected cell extracts (heat inactivated)
- HSV‐1 virus, cell‐free (heat inactivated)
- *Candida albicans* extract

These assays were performed on all subjects in each of the groups on day 1. The subjects in group A, the frequent cold sore sufferers, were then treated with a single dose of SADBE topically on the arm after their blood collection on day 1. They returned for blood collections on days 15 and 57 to measure the same outcomes.

## 2 MATERIALS AND METHODS

This study was a clinical trial titled: “A Phase 1 Study of the Immune Response to Herpes Simplex Virus Type 1 (HSV‐1) and General Immune Health in Subjects Infected with HSV‐1” conducted at Prism Clinical Research, St. Paul, MN, USA, in accordance with the principles of the Declaration of Helsinki. The study protocol, the investigator's brochure, and other trial‐related information were approved by an independent Institutional Review Board. The study protocol was reviewed, approved, and registered at ClinicalTrials.gov under registration no. NCT03661541.

### 2.1 Subjects

Subjects ages 18–64 who were positive for anti‐HSV‐1 IgG were recruited in three groups of 12 subjects each. The groups were approximately age matched and gender composition matched. The groups self‐reported different numbers of herpes labialis episodes over the prior 12 months: (A) 6 or more herpes labialis outbreaks over the previous 12 months, (B) 1 or 2 herpes labialis outbreaks over the previous 12 months, and (C) zero herpes labialis outbreaks over the previous 12 months. Group A was recruited first, and groups B and C were then recruited to be approximately sex and age matched to group A.

### 2.2 Procedures

After a screening visit at which blood was drawn to test for IgG antibody against HSV‐1 and subjects were interviewed for inclusion/exclusion criteria, selected subjects returned for blood draws on day 1. After the blood draw on day 1, subjects in group A only were dosed with SADBE. A petrolatum donut was applied with a cotton swab to form about a 1 cm diameter donut on skin on the inner aspect of the upper arm. Then a separate cotton swab was dipped in a 2% SADBE solution (w/v) in DMSO, and the swab was then used to apply about 10–20 mg of solution over about a 1 cm diameter circle within the petrolatum donut. Immediately after application, the application site was covered with TEGADERM. Subjects were advised to remove the TEGADERM and rinse and wipe the spot 3 h later. The group A subjects then returned for blood draws on days 15 and 57 and were queried about adverse events on those dates.

At this study visit where blood was drawn it was tested for blood cell counts, various cytokine levels, and anti‐HSV‐1 IgG quantitative levels.

Anti‐HSV‐1 IgG was quantitated with Focus Diagnostics HerpeSelect 1 ELISA IgG assay. Cell counts were obtained by flow cytometry with Miltenyi Biotec 7‐Color Immunophenotyping Kit Human. Plasma cytokines were quantitated with the Invitrogen ProcartaPlex Custom Multiplex Immunoassay using magnetic microsphere technology.

Blood was also collected for isolation of peripheral blood mononuclear cells (PBMC) and the PBMC were subsequently isolated the same day and plated the same day for proliferation assays and gene expression assays as described below.

PBMC were isolated using SepMate PBMC isolation tubes (Stem Cell Technologies, Vancouver, Canada) and Ficoll‐Paque according to the manufacturer's instructions. PBMC were isolated and suspended in negative control medium at 2 million cells/mL. PBMC suspension (100 μL) was added to 100 μL of medium in quadruplicate in 96‐well plates that had been prepared in advance and stored frozen at −70°C and thawed immediately before cell addition. After cell addition to the plates, the plates were immediately incubated at 37°C. The wells were in these final concentrations after addition of the 100 μL of PBMC suspension in negative control medium:

1. Negative control in medium (RPMI with glutamine and pen/strep, supplemented with 10% human AB serum).
2. Medium plus 16 μg/mL protein from heat‐inactivated HSV‐1‐infected Vero cell extracts.
3. Medium plus heat‐inactivated HSV‐1 cell‐free virus at a final 7.5 million pfu/mL.
4. Medium plus *Candida* cell extract. (Greer item number M15A50, 20 000 pnu/mL allergenic extract mixed molds *Candida albicans*, was diluted 1/50 into medium).
5. Positive control, medium plus 10 μg/mL concanavalin A.

Two plates were created for each subject at each collection point. One plate was incubated for 2 days at 37°C in a humidified 5% CO_2_ atmosphere and then RNA was isolated from the pooled contents of the four wells of a given condition for a specific patient, and the RNA was stored at −70°C and subsequently used for quantitative real time PCR analysis of gene expression of 41 immune‐related genes.

A second plate was incubated for 4 days. On the 4th day 1 μCi of tritiated thymidine in 50 μL of fresh negative control medium was added to each well. After 22 h incubation the cells were harvested onto a filter mat and each well counted by scintillation counting for tritium incorporation into DNA as a quantitative measure of cell division.

### 2.3 HSV‐1‐infected cell extracts

HSV‐1‐infected cell extracts were made by plating 10 million Vero cells in each of three T‐75 flasks on day 0, withdrawing all medium and adding cell‐free HSV‐1 virus (KOS strain) (ATCC strain VR‐1493) in 2 mL medium on day 1 (at a multiplicity of infection of about 3), incubating for 2 h to allow infection, and then adding 13 mL of medium. Then on day 2 cells were scraped off with cell scrapers, pelleted by low‐speed centrifugation, washed twice in PBS, resuspended in 2 mL water total for all three flasks pooled in one snap‐cap tube, and sonicated in a bath type sonicator in an ice‐water bath for 4 min. The resultant cell extracts were stored at −70°C.

### 2.4 Cell‐free HSV‐1 virus

Cell‐free HSV‐1 virus was made by plating 10 million Vero cells per T‐75 flask on day 0. On day 1 medium was removed, and about 10 million virus was added in 2 mL of medium per flask for a multiplicity of infection of about 1. This was incubated with rocking every 2 min for 1–2 h to allow viral adsorption to the cells. On day 4, the flasks were shaken to detach cells, the contents were then withdrawn and centrifuged at 1400*g* for 10 min to pellet cells and cell debris. The supernatant was collected and centrifuged at 23 000g for 2 h to pellet virus. The pellet was resuspended in 1 mL of Vero medium for each T‐75 harvested, and then frozen at −70°C. Virus prepared this way was found to have about 2.4 × 10^9^ pfu/mL.

Both the HSV‐1‐infected cell extracts and the cell‐free HSV‐1 virus were heated at 70°C for 30 min before adding them to the PBMC plates to heat inactivate virus to prevent it from killing the PBMCs in the plates.

### 2.5 HSV‐1 pfu determination

To determine the number of plaque forming units of HSV‐1 per mL in a preparation, the following assay was done. Vero cells were plated at 5000 cells per well in a 96‐well plate on day 0. On day 1 the HSV‐1 preparation was diluted in a series of 2.5‐fold dilutions from a 10 000‐fold dilution. Forty μL of each of the dilutions was added to wells in triplicate, and the plate incubated 6 more days at 37°C in a 5% CO_2_ humidified atmosphere. Then 50 μL of a 4:1 mixture of medium and Dojindo cell counting kit reagent was added to each well, and the absorbance at 450 nm read until the O.D. in negative control wells with no virus added reached 2.5. The greatest dilution with loss of more than 50% of cell viability in wells was considered to have 1 pfu added to the well.

### 2.6 Real time PCR

#### 2.6.1 RNA isolation and pre‐amplification

As noted above, after 2 days of growth of the PBMC in a 37°C humidified 5% CO_2_ incubator in a 96‐well plate, four wells of a single condition (negative control, positive control, heat‐inactivated HSV‐1, HSV‐1‐infected cell extracts, or *Candida* extract) with cells from a single subject (200 μL in each well) were pooled, and the RNA was immediately isolated with Trizol reagent and chloroform by Qiagen RNeasy MiniElute clean up kit Qiagen cat #74204, according to manufacturer's instructions into a final volume of 13 μL, and then flash frozen on powdered dry ice, and stored at −70°C in aliquots of 3 μL for RNA quantification and 10 μL for PCR analysis.

RNA was preamplified with the Qiagen RT2 PreAMP cDNA synthesis kit (Ref. 330451) and custom RT2 PreAmp primer mix (Ref. 330141) starting with about 50 ng RNA according to the procedure of RT preamp cDNA synthesis handbook date 03/2011, pages 17‐20. The amplification reactions (final 120 μL) were frozen at −70°C and used for qPCR analysis the next day.

#### 2.6.2 qPCR

qPCR was done with the Qiagen RT PCR profiler gene analysis kit (catalog number 330451) using RT2 Sybr Green Rox qPCR Mastermix (24) (Part 330523) and Custom RT2 PCR array plates (Ref. 330171). For this 675 μL 2× RT2 SYBR green mastermix, 51 μL pre‐AMP synthesis reaction, and 623 μL RNase free water were mixed, and then 25 μL of the mixture was added to 48 wells on the left or right half of the custom PCR plate. The PCR instrument was Applied Biosystems 7900HT fast real time PCR. The program was 95°C for 10 min; then 40 cycles of 95°C for 15 s, 60°C for 1:00; then stage 3 denaturation stage of 95°C for 15 s, 60°C for 15 seconds, and 95°C for 15 s. The detector and reporter dye was Sybr, passive reference was Rox. For data analysis a manual Cq of 0.5 was used for all runs.

Control genes were ACTB, RPLP0, GAPDH. There were 41 test genes involved in immune function, and three wells for reverse transcriptase control, genomic DNA control, and positive PCR control. A fixed threshold level of 0.5 was used for all plates, and the threshold cycle (Ct) was calculated for each well as the cycle when the signal crossed the threshold.

ΔCt for each test gene was Ct of the test gene minus average Ct of the three housekeeping genes in the same well. Statistical significance of differences in absolute expression levels was calculated based on ΔCt.^13^, ^14^ Average absolute gene expression was calculated as 2 ^‐ average ΔCt^.^15^ ΔΔCt was calculated as ΔCt in a stimulus (eg, heat‐inactivated HSV‐1) minus ΔCt for the same individual at the same collection time point in negative control medium. Individual fold‐regulation or fold‐stimulation by the stimulus was 2^‐ΔΔCt^.^15^ Statistical significance of differences in fold‐regulation or fold‐stimulation was calculated based on ΔCt in the stimulus versus ΔCt in the negative control medium. Average fold regulation was 2^‐ average ΔΔCt^.

### 2.7 Statistics

*P* values for comparisons between group A and groups B or C were calculated using unpaired 2‐tailed Student's *t*‐test, and between group A on day 1 versus days 15 or 57 using the paired 2‐tailed Student's *t*‐test.

## 3 RESULTS

### 3.1 Subjects

The summary statistics of the subjects are in Table 1. There were 12 subjects in each group.

**Table 1:** Subject demographics

Group	Median Age	Age range	Sex
A (6 or more outbreaks)	54.06	28.3 to 60.4	9F/3M
B (1 or 2 outbreaks)	54.06	28.1 to 61.1	9F/3M
C (0 outbreaks)	54.30	32.2 to 60.3	8F/4M

### 3.2 Adverse events

The study drug was well tolerated. Of the 12 subjects treated, two experienced stinging at the application site, and one had nausea that was ruled possibly related to the study drug. No rashes or delayed‐type hypersensitivity reactions were reported, which is sometimes seen with SADBE.

### 3.3 PBMC Proliferation in vitro

**Figure 1:**
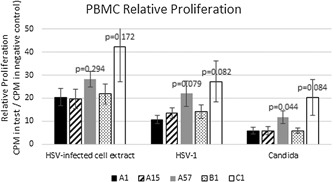
PBMC relative proliferation (CPM in medium with the test stimulus/CPM in negative control (medium alone). *P* values are for the indicated group/day versus A1 in the same stimulus. (*n* = 12.)

The relative proliferation for each test condition was calculated as (avg. CPM test condition/avg. CPM negative control). The average CPM is the average tritium counts per minute of the four wells in a given test condition for a given collection point of a given patient. Table 2 and Figure 1 show the results. The test condition one of the three antigenic stimuli—HSV‐1‐infected cell extracts, HSV‐1 virus, and *Candida*—or the positive control stimulus concanavalin A; and the negative control is the medium‐only unstimulated condition.

**Table 2:** Proliferation (±SEM) of PBMCs collected from frequent cold sore sufferers (group A), infrequent cold sore sufferers (group B), and persons with zero cold sores in the prior 12 months (group C)

		Stimulus					
Group and day		Negative control (media only)	HSV‐infected cell extract	HSV‐1	Candida	Concanavalin A positive control	All 3 test conditions, normalized as percent of C1 averages
A1	CPM	719 (±137)	13240 (±2621)	7134 (±1431)	4016 (±1145)	83020 (±10486)	
	Relative proliferation		20.36 (±3.65)	10.63 (±1.74)	5.79 (±1.46)	36.78 (±20.27)	38.67% (±4.41%)
A15	CPM	719 (±46)	15206 (±3739)	10086 (±2038)	4533 (±1483)	81524 (±11272)	
	Relative proliferation		19.66 (±4.23)	13.35 (±2.24)	5.8 (±1.79)	111.46 (±11.70)	41.47% (±5.31%)
A57	CPM	701 (±139)	15651 (±2811)	12635 (±2516)	7349 (±2078)	96986 (±11468)	
	Relative proliferation		28.20 (±6.49)	21.92 (±5.53)	11.84 (±2.75)	225.23 (±66.92)	68.70% (±9.49%)
	*P*‐value vs A1		*P* = 0.249	*P* = 0.079	*P* = 0.044	*P* = 0.211	*P* = 0.005
B1	CPM	770 (±62)	16147 (±2956)	10824 (±2279)	4791 (±1204)	93337 (±11452)	
	Relative proliferation		21.84 (±4.42)	14.12 (±2.82)	5.85 (±1.21)	128.56 (±19.69)	44.23% (±5.46%)
C1	CPM	629 (±99)	17036 (±2895)	11344 (±1960)	8588 (±2743)	103421 (±12809)	
	Relative proliferation		42.22 (±15.06)	27.13 (±8.89)	20.25 (±7.86)	241.98 (±55.25)	100.00% (20.09%)
	*P*‐value vs A1		*P* = 0.172	*P* = 0.082	*P* = 0.084	*P* = 0.094	*P* = 0.004

Relative proliferation is CPM in the test stimulus/CPM in negative control (medium only). *P* values are for Relative Proliferation in the test condition versus A1, by paired *t*‐test for A57 versus A1 and unpaired *t‐*test for C1 versus A1.

**Figure 2:**
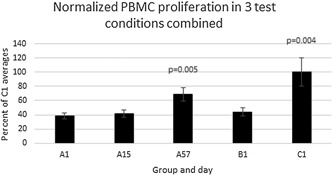
PBMC normalized proliferation in the three test stimuli taken together (HSV‐1‐infected cell extracts, heat‐inactivated HSV‐1, and *Candida* extract). Normalized proliferation values are the individual relative proliferation value in a given test stimulus as a percent of the C1 relative proliferation group average in that stimulus. *P* values are for the indicated group/day versus A1

On day 1 group C (zero outbreaks) had substantially higher proliferation in all three test conditions and in the concanavalin A positive control. The differences vs group A1 (group A day 1) were almost significant (between 0.05 and 0.10) for HSV‐1, *Candida*, and Concanavalin A. In group A, after treatment on day 1, proliferation in the three test conditions and in Con A positive control was basically unchanged on day 15 but was substantially increased on day 57 in all three test conditions and in Con A positive control. The difference between A1 and A57 by paired *t*‐test was significant for *Candida* and almost significant for HSV‐1. A paired *t*‐test was done between the day 57 and day 1 combined relative proliferation values in all of the three test antigens (HSV‐1‐infected cell extracts, HSV‐1 virus, and *Candida*), and the *P*‐value was 0.0109 (*n* = 36, since there were 3 values for each of 12 individuals). However, to perform a similar comparison between groups C and A1, the relative proliferation values in the three test antigens needed to be normalized because different stimuli were used and the average proliferation was different in the different stimuli. So the relative proliferation for each individual in each stimulus was normalized to a percentage of the average relative proliferation in group C. An unpaired *t*‐test of normalized relative proliferations in the three specific stimuli in group C versus group A1 (*n* = 36) give *P* = 0.0039. When the paired *t*‐test is done with the normalized relative proliferation of group A57 versus A1, *P* = 0.0047. So proliferation in the three test antigens taken together is higher in group C than A1 and in group A day 57 than A day 1, and in both cases the difference is highly significant (*P* < 0.01). (Table 2 and Figure 2).

We wondered whether the increased proliferation that we saw in HSV‐1‐infected Vero cell extracts was due to the HSV‐1 proteins or the Vero cell proteins, so we tested proliferation of PBMCs from two donors in group C with uninfected Vero cell extract as well. In those two patients the average cpm in medium‐alone negative control, uninfected Vero cell extracts, and HSV‐1‐infected Vero cell extracts were respectively 300, 526, and 6213 for the first patient and 456, 438, and 12652 in the second patient. So for both patients the proliferation with uninfected Vero cell extracts was essentially the same as in medium‐alone negative control and was very much less than proliferation in HSV‐1‐infected cell extracts. From this, we can conclude that the proliferation we saw with HSV‐1‐infected Vero cell extract was due to stimulation of PBMCs by the HSV‐1 proteins, not by the Vero proteins.

### 3.4 Blood cell counts, plasma cytokine levels, and serum anti‐HSV‐1 IgG levels

#### 3.4.1 Blood cell counts

Certain blood cell count parameters were measured in the study—specifically, absolute counts of natural killer cells, B cells, T cells, CD4 cells, CD8 cells, lymphocytes, and percentages and ratios of the above. Almost none of the measures were significantly different between the different groups or on day 15 or 57 versus day 1 in group A. The data where statistical significance was found and helper/cytotoxic ratio (CD4/CD8) where the result was not significant but a possible pattern of lower ratios in groups B and C versus A1 and in A15 and A57 versus A1 was found are shown in Table 3.

**Table 3:** Selected blood cell counts of subjects

Units	Group and day	Group average	Group St. Dev.	*P‐*value* vs A1	Sample minus A1	Sample/A1
Absolute Count CD19 (B‐Cells)						
Cells/mm^3^	A1	194.37	79.93			
Cells/mm^3^	A15	**171.74**	85.20	**0.033**	−22.63	0.88
Cells/mm^3^	A57	192.52	74.12	0.885	−1.85	0.99
Cells/mm^3^	B1	166.18	84.67	0.411	−28.19	0.85
Cells/mm^3^	C1	202.72	88.72	0.811	8.35	1.04
Helper/Cytotoxic Ratio						
	A1	5.25	5.41			
	A15	3.58	1.82	0.306	−1.67	0.68
	A57	4.35	3.05	0.331	−0.90	0.83
	B1	3.07	0.93	0.182	−2.18	0.58
	C1	3.15	0.94	0.199	−2.10	0.60
Percentage CD19 (B‐Cells)						
%	A1	12.25	3.51			
%	A15	**10.17**	3.91	**0.004**	−2.08	0.83
%	A57	11.67	4.03	0.253	−0.58	0.95
%	B1	12.08	4.27	0.918	−0.17	0.99
%	C1	12.25	3.61	1.000	0.00	1.00
Percentage CD3 (T‐Cells)						
%	A1	76.08	6.63			
%	A15	77.50	5.32	0.215	1.42	1.02
%	A57	**78.67**	4.55	**0.037**	2.58	1.03
%	B1	71.67	5.59	0.091	−4.42	0.94
%	C1	77.83	4.41	0.454	1.75	1.02
T‐cell to B‐cell ratio						
	A1	6.81	2.41			
	A15	**9.22**	4.86	**0.023**	2.41	1.35
	A57	7.50	2.56	0.099	0.69	1.01
	B1	7.13	3.95	0.813	0.32	1.05
	C1	7.13	2.77	0.762	0.32	1.05

* *P* values are from two‐tailed unpaired *t*‐test for groups B and C versus A and from two‐tailed paired *t*‐test for days 15 and 57 versus day 1 in group A. *n* = 12 in each group.

#### 3.4.2 Cytokines

Plasma cytokine levels of the following cytokines were measured: IL‐gamma, IL‐1‐beta, IL‐10, IL12/IL23 p40, IL‐13, IL17A, IL‐4, IL‐5, IL‐6, IL‐8, tumor necrosis factor alpha, IL‐2, and IL‐2 receptor. For all but IL‐2 and IL‐2R the majority of subjects had undetectable levels. There were no significant differences between groups or on days 15 or 57 versus day 1 in group A. (Data not shown).

#### 3.4.3 Anti‐HSV‐1 IgG

**Figure 3:**
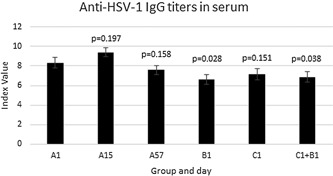
Anti‐HSV‐1 IgG levels in serum by group and day ±SEM. *P* values are for the indicated group/day versus A1

Anti‐HSV‐1 IgG was quantified in serum collected at each study visit. The levels are shown in Figure 3. An index value of >1.0 is considered positive for anti‐HSV‐1 IgG.

Group B had significantly lower anti‐HSV1 IgG antibody than group A at day 1 and groups B and C pooled together had a significantly lower level of anti‐HSV1 IgG than group A at day 1. Group C also had a lower level of anti‐HSV1 antibody than group A, although the difference was not statistically significant. And on day 57, group A's anti‐HSV1 IgG level was lower than it had been on day 0 (*P* = 0.16), which is a move by day 57 toward the characteristic of the groups with fewer herpes labialis outbreaks. Since the antibody level initially went up in group A from day 1 to day 15, the change from day 15 to day 57 in group A was a highly significant decrease in anti‐HSV1 IgG (*P* < 0.01).

### 3.5 Cytokine and immune‐related gene expression

qRT‐PCR was conducted on RNA collected from cells after 2 days of culture in medium with no stimulus (negative control) or with HSV‐1‐infected cell extracts, heat‐inactivated HSV‐1 virus, or with *Candida* fungal extract.

**Figure 4:**
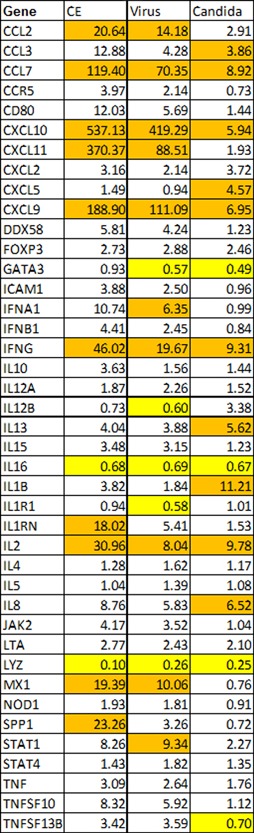
Average fold‐regulation of immune‐related genes from PBMCs of group B and C subjects pooled. The 10 most highly up‐regulated genes for each stimulus are in orange highlight and the genes down regulated at 0.70 or below are in yellow highlight. Fold‐regulation is absolute gene expression in the stimulus divided by absolute gene expression in the negative control

Figure 4 shows the average fold regulation of each gene in the groups B and C subjects combined, those with zero or few outbreaks and thus better immune control of HSV‐1. Fold‐regulation is absolute gene expression in the stimulus divided by absolute gene expression in the negative control.

Almost all the gene expression changes shown in Figure 4 are statistically significant (*P* < 0.05) and in particular all of the changes highlighted in either yellow or orange are statistically significant.

The two Th1 cytokines^16^, ^17^—IFNG and IL2—are both among the most upregulated genes in all three stimuli, while the Th2 cytokines^16^, ^17^ tested—IL4, IL5, IL10, and IL13—are much less upregulated by the stimuli, if upregulated at all.

#### 3.5.1 Differences in PBMC gene expression between groups with frequent, infrequent, or no herpes labialis outbreaks (groups A, B, and C), and difference in PBMC gene expression in group A after SADBE treatment

**Figure 5:**
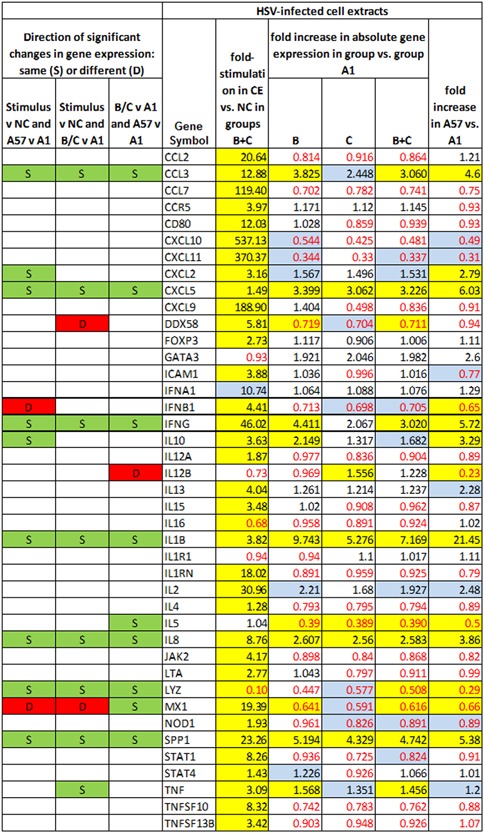
Comparison of changes or differences in gene expression of PBMCs in vitro stimulated with HSV‐1‐infected cell extracts in groups B+C in stimulus versus negative control (NC) (first column to right of genes); between groups B, C, or B+C versus A day 1 (2nd‐4th columns to right of genes), and in group A day 57 versus day 1 (far right column). Significant differences (*P* < 0.05) are highlighted in yellow; 0.20>*P*>0.05 in blue. Decreased expression (ratio less than 1.0) in any gene expression ratio is shown in red type. The left three columns show, for cases where there is a significant difference in gene expression (1) in the stimulus versus negative control; (2) in group C or B+C versus A1; and (3) in group A day 57 versus group A day 1, whether pairs of those significant differences are in the same direction (*i.e*., both increases or both decreases in gene expression) (S highlighted in green) or different directions (D highlighted in red)

**Figure 6:**
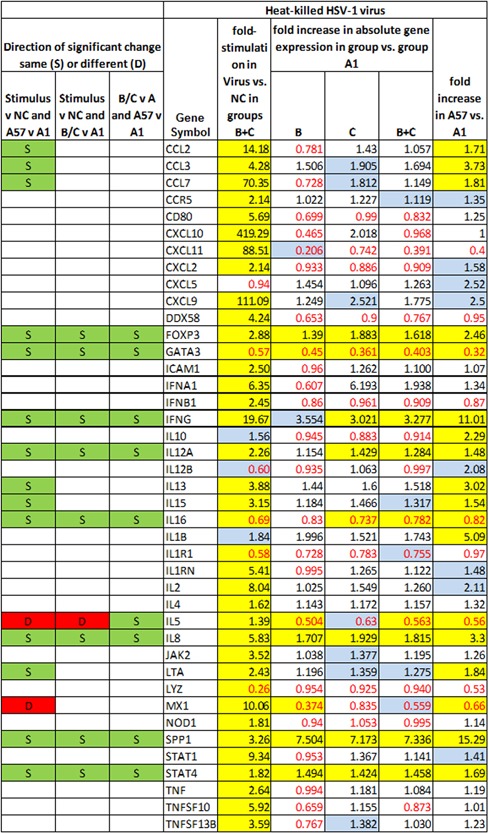
Comparison of changes or differences in gene expression of PBMCs in vitro stimulated with heat‐inactivated HSV‐1 virus in groups B+C in stimulus versus negative control (NC) (first column to right of genes); between groups B, C, or B+C versus A day 1 (2nd‐4th columns to right of genes), and in group A day 57 versus day 1 (far right column). Significant differences (*P* < 0.05) are highlighted in yellow; 0.20>*P*>0.05 in blue. Decreased expression (ratio less than 1.0) in any gene expression ratio is shown in red type. The left three columns show, for cases where there is a significant difference in gene expression (1) in the stimulus versus negative control; (2) in group C; or B+C versus A1; and (3) in group A day 57 versus group A day 1, whether pairs of those significant differences are in the same direction (*i.e*., both increases or both decreases in gene expression) (S highlighted in green) or different directions (D highlighted in red)

**Figure 7:**
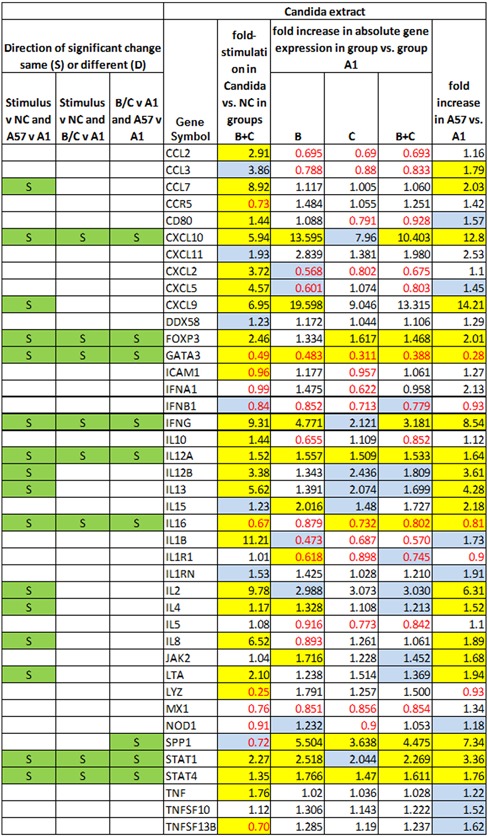
Comparison of changes or differences in gene expression of PBMCs in vitro stimulated with Candida extract in groups B+C in stimulus vs. negative control (NC) (first column to right of genes); between groups B, C, or B+C versus A day 1 (2nd‐4th columns to right of genes), and in group A day 57 versus day 1 (far right column). Significant differences (*P* < 0.05) are highlighted in yellow; 0.20>*P*>0.05 in blue. Decreased expression (ratio less than 1.0) in any gene expression ratio is shown in red type. The left three columns show, for cases where there is a significant difference in gene expression (1) in the stimulus versus negative control; (2) in group C or B+C versus A1; and (3) in group A day 57 versus group A day 1, whether pairs of those significant differences are in the same direction (*i.e*., both increases or both decreases in gene expression) (S highlighted in green) or different directions (D highlighted in red)

**Figure 8:**
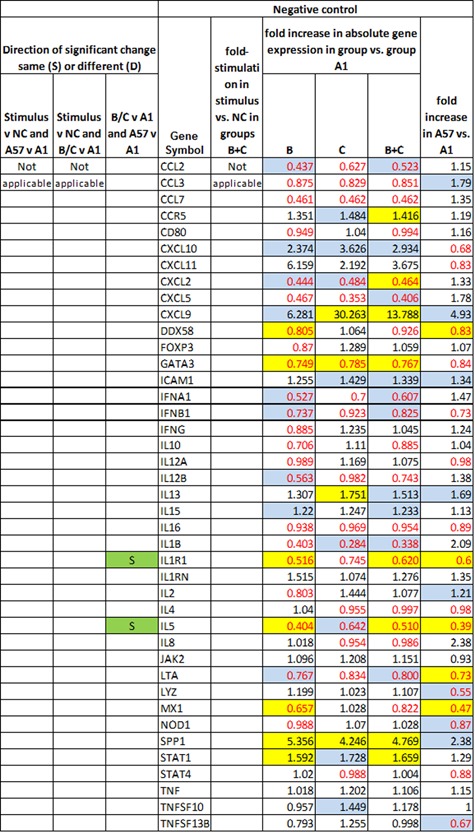
Comparison of differences in gene expression of PBMCs in vitro in negative control medium (NC) between groups B, C, or B+C versus A day 1 (2nd‐4th columns to right of genes), and in group A day 57 versus day 1 (far right column). Significant differences (*P* < 0.05) are highlighted in yellow; 0.20>*P*>0.05 in blue. Decreased expression (ratio less than 1.0) in any gene expression ratio is shown in red type. The left three columns show, for cases where there is a significant difference in gene expression (1) in the stimulus versus negative control; (2) in group C or B+C versus A1; and (3) in group A day 57 versus group A day 1, whether pairs of those significant differences are in the same direction (*i.e*., both increases or both decreases in gene expression) (S highlighted in green) or different directions (D highlighted in red)

Figures 5, 6, 7, 8 show several aspects of PBMC gene expression in vitro in each of the three stimuli and negative control. To the right of the gene symbol, the first column is the fold stimulation of the gene in the stimulus/negative control medium, the same information shown in Figure 4. The next three columns to the right are the ratio of absolute gene expression in groups B, C, or B+C divided by that in group A day 1 (A1). The right‐most column is the ratio of absolute gene expression in group A day 57/day 1. Decreased expression (ratio less than 1.0) in any expression ratio is shown with red type.

The three columns to the left of the gene symbol in Figures 5–8 indicate the comparison of direction of change or difference in any significant differences in gene expression. Only statistically significant results are included. In each column, two types of differences are compared, and if the direction of both differences is the same (both are increases or both are decreases in gene expression) it is marked S and highlighted in green; if the directions of the two changes are different, it is marked D and highlighted in red. The changes compared are (1) (stimulus vs negative control) compared to (group A day 57 vs A day 1), (2) (stimulus vs negative control) compared to (groups C or B+C vs. group A day 1), and (3) (groups C or B+C vs group A day 1) compared to (group A day 57 vs A day 1).

**Figure 9:**
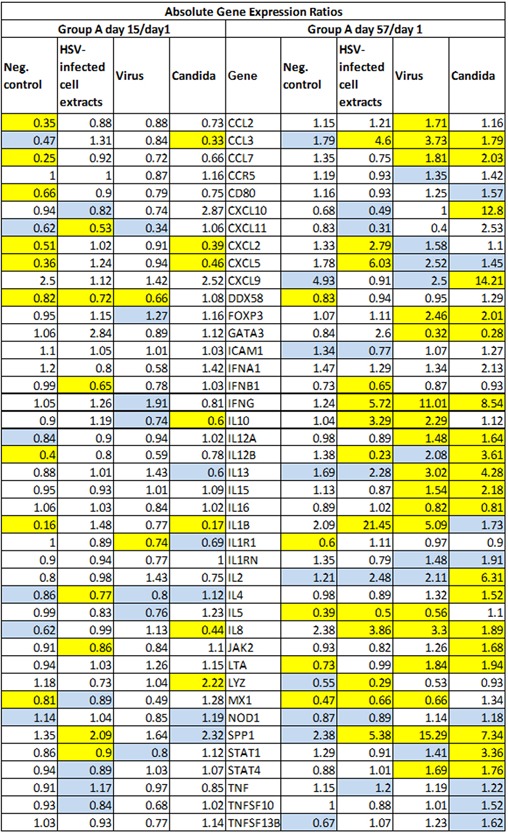
Ratios of PBMC absolute gene expression in group A on days 15 and 57 compared to day 1. Significant differences (*P* < 0.05) are highlighted in yellow; 0.20>*P*>0.05 in blue

Figures 5‐8 show the ratio of PBMC absolute gene expression in group A on day 57 to day 1 but not group A day 15. The gene expression in group A on day 15 is not shown because there were far more significant differences in gene expression on day 57 versus day 1 than on day 15 versus day 1 and the differences were generally larger (Figure 9).

Figure 5 concerns the HSV‐1‐infected cell extract as stimulant, Figure 6 heat‐inactivated HSV‐1 virus, Figure 7 *Candida* extract, and Figure 8 negative control medium only.

IFNG was significantly more expressed in groups B+C combined than group A day 1 in all three stimuli. The other genes besides IFNG significantly more expressed in groups B+C than A in HSV1‐infected cell extracts were CCL3, CXCL5, DDX58, IL1B, IL8, SPP1, and TNF. In HSV‐1 virions they were FOXP3, IL12A, IL8, SPP1, and STAT4; and in *Candida* extract they were CXCL10, FOXP3, SPP1, STAT1, and STAT4. The genes significantly less expressed in groups B+C than A in HSV‐1 infected cell extracts were DDX58, IL5, LYZ, and MX1; in HSV‐1 virions the genes less expressed in groups B+C than A were GATA3, IL16, and IL5; and in *Candida* extract they were GATA3 and IL16.

Many genes had significant changes in gene expression in group A on day 57 compared to day 1 in the three stimuli, and in essentially every case the same gene's expression was also changed in the same direction in groups B+C compared to A1. In fact, for *every* gene with a significant change in A57 versus A1 and in group B+C combined versus A1 the change was in the same direction.

In the unstimulated negative control condition (Figure 8) the only genes with a significant difference in gene expression in both (groups B+C vs group A day 1) and (group A day 57 vs day 1) were IL1R1 and IL5, and both of those genes was less expressed in groups B+C than in A and in group A day 57 than on day 1 in the unstimulated negative control condition. So here also, all changes in gene expression were in the same direction to make group A on day 57 more like those with better immune control of the HSV‐1.

#### 3.5.2 Changes in gene expression in group A from day 1 to days 15 and 57

The ratios of day 15/day 1 and day 57/day 1 PBMC absolute gene expression in group A are shown in Figure 9. In the three stimulated conditions, there were far more significant differences in gene expression on day 57 than on day 15 compared to day 1, and that is why the day 57 data only is used in Figures 5–8. This is consistent with the PBMC proliferation data that shows essentially no change in proliferation at day 15 but an increase in proliferation in all three stimuli on day 57.

#### 3.5.3 Group A goes from lower IFNG expression than groups B+C on day 1 to higher than groups B+C on day 57 in all three stimuli

Table 4 shows how the expression of the IFNG gene in group A on days 1 and 57 compares to groups B+C pooled. At day 1, group A's expression of IFNG was significantly less than groups B+C pooled in all three stimuli. At day 57, it was above that of groups B+C in all three stimuli, and was significantly more than groups B+C in heat‐killed HSV virus. So with the HSV‐1 virus stimulus, expression of IFNG in the frequent herpes labialis sufferers goes from significantly less than groups B+C to significantly more than them 56 days after treatment with SADBE. Thus, SADBE treatment does not just partially reduce the defect in IFNG expression in those with frequent herpes labialis episodes, it completely reverses it.

**Table 4:** Ratio of expression of the IFNG gene in group A on days 1 and 57 versus Groups B+C pooled

	Ratio of absolute IFNG gene expression in group A vs Groups B+C pooled			
	Neg Control	HSV‐infected cell extracts	Virus	Candida
Group A, day 1	0.957	0.331*	0.305*	0.314*
Group A day 57	1.190	1.896	3.359*	2.684

* Statistically significant difference versus B+C pooled, *P *< 0.05

## 4 DISCUSSION

The data here show a cellular immune response is more important than humoral and a Th1 immune response more important than Th2 in controlling HSV‐1 outbreaks as herpes labialis. And the data show that a single dose of SADBE changes the immune response of subjects with frequent herpes labialis over 8 weeks to make it more like, and at least in IFNG expression better than, the immune response of subjects with few or no herpes labialis outbreaks.

### 4.1 Regulation of gene expression by the HSV and fungal stimuli

The gene regulation is somewhat more similar between HSV‐1‐infected cell extracts and HSV‐1 virus than between either HSV‐1‐infected cell extracts and *Candida* fungal extract or HSV‐1 virus and *Candida* (Figure 4). But for all three stimuli interferon gamma (IFNG) is among the most up‐regulated genes. IL2, another Th1‐produced cytokine, is also up regulated strongly with all three stimuli. The up‐regulation was generally lesser in *Candida* extract than with HSV‐infected cell extract or virus, possibly because the *Candida* components were not at a high concentration in the wells. CXCL9, 10, and 11 are all very highly upregulated with the two HSV stimuli and 9 and 10 were rather upregulated by *Candida* as well. Those genes are all induced by IFNG.^18^ CCL7 is also strongly upregulated by all three stimuli.

LYZ (lysozyme gene) is down‐regulated by all three stimuli. That makes sense because it is an antibacterial enzyme and the three stimuli are viral and fungal.

MX1 is a gene involved in antiviral response,^19^ and it is strongly upregulated by both HSV stimuli and down‐regulated by *Candida*.

SPP1 is strongly upregulated by HSV‐1‐infected cell extracts, and to a lesser extent by virus, but down regulated by *Candida*. SPP1 is a cytokine that upregulates expression of IFNG and IL12. It is also a regulator of apoptosis.^20^ Apoptosis of viral‐infected cells is a key method of immune control of viral infections, which may explain why SPP1 is upregulated by the viral stimuli but not by *Candida* extract.

GATA3 is significantly down regulated by virus and *Candida* and slightly down regulated by HSV‐1‐infected cell extracts. GATA3 induces differentiation of Th0 cells to the Th2 subtype and suppresses differentiation to the Th1 subtype.^21^, ^22^ Since it is down regulated by these stimuli, the stimuli tend to drive T cells to the Th1 subtype.

IL16 is down‐regulated by all three stimuli.

### 4.2 Differences in groups B and C versus A day 1 and in group A on day 57 versus day 1

#### 4.2.1 PBMC proliferation in vitro to HSV‐1 and other stimuli

PBMC proliferation in response to all three stimuli showed the same trends of group C1>B1>A1. (In raw CPM, group B1 was about midway between groups C1 and A1 in all three stimuli, while by relative proliferation (relative to negative control) it was closer to group A1). In other words, PBMC from those with better immune control of HSV‐1 proliferated more than those with worse immune control. The difference was not statistically significant for any one stimulus (*P* = 0.08 for HSV‐1 virus and *P* = 0.08 for *Candida*, *n* = 12), but the response to all three stimuli taken together and normalized was highly significantly greater in group C than group A (*P* < 0.01, *n* = 36).

PBMC proliferation against all three test stimuli for the group A subjects was also greater on day 57 than on day 1 before drug treatment, significantly so for *Candida* (*P* = 0.08 for HSV‐1 and *P* = 0.044 for *Candida*, *n* = 12). The PBMC proliferative response to all three stimuli normalized and taken together was significantly greater on day 57 than day 1 in the group A subjects (*P* < 0.01, *n* = 36)). Thus, in regard to proliferation of PBMCs in vitro against HSV‐1‐infected cell extracts, HSV‐1 virus, and *Candida* extract, the group A subjects were more like the group C subjects with good immune control of HSV infection 56 days after SADBE treatment than they had been on day 1 before drug treatment.

#### 4.2.2 Anti‐HSV‐1 IgG

Groups C and B pooled together had significantly lower anti‐HSV‐1 IgG levels than group A. Among group A subjects, anti‐HSV‐1 IgG levels were lower on day 57 than day 1, although not significantly so, making the group A subjects again more like those with fewer outbreaks and better immune control of the virus on day 57 than they had been on day 1.

#### 4.2.3 Immune gene expression

For almost every immune‐related gene where any differences were significant the direction of these changes match:

1. Expression in PBMCs upregulated (or downregulated) by HSV‐1 (or other stimulus).
2. Same upregulated gene was more expressed in the presence of HSV‐1 (or other stimulus) in group C and B subjects (those with zero or infrequent herpes labialis episodes) than group A subjects (those with frequent herpes labialis episodes). (If the gene was downregulated by the stimulus, then it was also found to be less expressed in groups C and B than group A).
3. Same upregulated gene was more expressed in the presence of HSV‐1 (or other stimulus) in group A subjects on day 57 after treatment with SADBE than on day 1 before treatment. (If the gene was downregulated by the stimulus, then it was also found to be less expressed in group A on day 57 than day 1).

Thus in essentially every case, the SADBE treatment changed the group A subjects by day 57 to make them much more like the group B and C subjects who have better immune control of their HSV‐1 infection than the group A subjects were on day 1.

**Figure 10:**
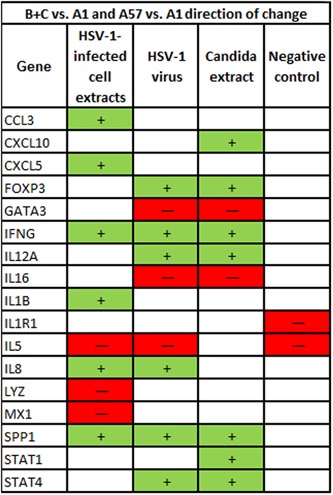
List of every gene where both groups B+C vs. A1 and A57 vs. A1 had significant differences of absolute gene expression in a given condition, and the direction of the difference relative to A1. In every case, the direction of the difference was the same for B+C vs. A1 and A57 vs. A1—both higher than A1 (+) or both lower than A1 (−)

Figure 10 lists every gene that has a significant difference in gene expression in both groups B+C pooled versus A1 and in group A day 57 versus day 1 in the same condition. Seventeen genes are listed and a total of 29 gene‐condition combinations. In *every case*, the change in expression is in the same direction: that is, group A on day 57 becomes more like groups B and C than it had been on day 1. And in nearly every case genes with higher expression in groups B+C than A1 and in A57 than A1 were upregulated in the corresponding stimulus and those with lower expression were downregulated by the stimulus. The lone clear exception is MX1, which was less expressed in HSV‐1‐infected cell extracts in groups B+C versus A1 and in A57 versus A1 but was markedly upregulated by the stimulus (Figure 5).

IFNG (a Th1 cytokine) was the gene (1) most consistently upregulated by a large amount in all three stimuli versus negative control; (2) most consistently increased by the greatest amount in its expression in groups B+C versus group A day 1 in all three stimuli, and (3) most consistently increased in its expression by the greatest amount in group A day 57 versus day 1 in all three stimuli. IL5 (a Th2 cytokine) (1) was not markedly upregulated by any of the three stimuli and (2) was less expressed in groups B+C than group A day 1 in both HSV‐1 virus and HSV‐1‐infected cell extracts and (3) was less expressed in group A on day 57 than day 1 in both HSV‐1 virus and HSV‐1‐infected cell extracts.

These results are consistent with, and tend to confirm, the results of the prior clinical trial that showed a single topical application of 2% SADBE to the arm delayed next cold sore outbreak in persons with frequent cold sores.^9^ SADBE apparently takes more than 2 weeks to exert its effects on the immune system. The changes in both PBMC proliferation and immune gene expression were greater at 8 weeks after drug application than at 2 weeks after drug application. Interestingly, in the clinical trial there was also a suggestion in the data that SADBE did not exert its effect until more than about 3 weeks after drug application.^9^

Others have previously shown that CD8+ T cells are important in controlling herpes labialis outbreaks.^23^, ^24^, ^25^, ^26^, ^27^, ^28^, ^29^, ^30^, ^31^, ^32^ Our data are consistent with this. The helper/cytotoxic cell ratio (CD4+/CD8+ ratio) was 5.25 in group A versus 3.07 and 3.15, respectively in groups B and C (Table 3). The *P*‐value is less than 0.20 for comparisons of group A to both groups B and C, and if groups B and C are pooled and compared to group A the *P*‐value is 0.065, almost significant. This is consistent with prior evidence discussed below that CD8+ T cells are important and effective in controlling HSV recurrences.

Consistent with our finding *lower* anti‐HSV‐1 IgG levels correlate with better control of HSV‐1 outbreaks, Spruance et al. also found lower serum anti‐HSV‐1 antibodies in HSV seropositive patients with a history of frequent herpes labialis than in seropositive persons with no history of herpes labialis.^33^

Several prior reports also found IFN‐gamma to be important in controlling HSV infection and reducing HSV outbreaks. Dobbs et al^28^ showed that CD8+ T cells were able to clear an HSV‐2 infection in transgenic mice, but that efficacy was blocked in vivo by anti‐IFNG IgG. Liu et al^31^ showed that CD8^+^ T‐cells could prevent HSV‐1 reactivation from latency in excised trigeminal ganglia (TG), and that IFN‐gamma protein was produced by the CD8+ T cells, and that neutralization of IFN‐γ significantly enhanced the rate of HSV‐1 reactivation from latency in TG cultures. Spruance et al^33^ found that IFN‐gamma protein levels in PBMC supernatants stimulated with HSV‐1‐infected cell extracts were lower in frequent herpes labialis sufferers than HSV‐1 seropositive controls, consistent with the present result for IFNG gene expression in PBMC stimulated with heat‐killed HSV‐1. McKenna et al^7^ assayed IFN‐gamma in medium of PBMCs cultured in vitro and stimulated with inactivated HSV‐1 and found IFN‐gamma was at higher concentrations in medium of PBMCs from infrequent herpes labialis sufferers than frequent sufferers, also consistent with our findings. Cunningham et al^34^ showed higher interferon levels (including alpha, gamma, and lambda) in supernatants of PBMCs stimulated with heat‐killed HSV‐1 virus correlated with longer time to next herpes labialis recurrence. Carr et al^35^ showed that transgenic expression of IFNG could prevent HSV‐1 reactivation in a mouse model.

Our data are consistent with this in linking IFNG expression inversely with frequency of herpes labialis outbreaks. IFNG, the key Th1 cytokine, was at least nine‐fold overexpressed in groups B+C in the stimuli versus negative control, at least three‐fold more highly expressed in all three stimuli in groups B+C than group A on day 1, and at least eight‐fold more expressed in all three stimuli in group A on day 57 than day 1.

Conversely, IL5, a Th2 cytokine, was not markedly upregulated by any of the three stimuli versus negative control, was significantly less expressed in groups B+C than group A on day 1 in both HSV‐infected cell extracts and heat‐killed HSV‐1 virus, and in the negative control condition, and was significantly less expressed in both HSV‐infected cell extracts and HSV1‐ virus and in the negative control in group A on day 57 than on day 1. IL‐5 stimulates B cells and antibody production,^17^, ^36^ so the fact that its expression in the HSV‐1‐stimulated PBMCs is positively correlated with frequent recurrences is further evidence that antibody production is not important or is even detrimental for control of recurrences.

Thus, the two genes that appear to be most linked to immune prevention of HSV‐1 outbreaks but in opposite directions are IFNG (increased expression in both HSV stimuli) and IL5 (decreased expression in both HSV stimuli). This link of lower IL5 expression or protein level to reduced HSV‐1 recurrences has not previously been shown to our knowledge.

SADBE appears to somehow reset the immune system 8 weeks after drug application to more of a type 1 cellular immune response and a larger cellular immune response to the virus—with greater PBMC proliferation against heat‐killed HSV‐1, and greater IFNG expression and lower IL5 expression in response to both the heat‐killed HSV‐1 virus and HSV‐1‐infected cell extracts. These same differences characterize HSV‐1‐seropositive subjects with few or no herpes labialis outbreaks as opposed to those with frequent outbreaks, so SADBE resets the immune response to the virus to more like that of persons with more effective immune control of the virus.

Moreover, SADBE dosing did not just ameliorate the defect in group A immune response to HSV‐1, it completely reversed it in the key measure of IFNG expression in the presence of HSV‐1 virus: At day 1 group A had significantly lower IFNG expression than groups B+C, whereas at day 57 group A had significantly higher IFNG expression than groups B+C.

Interestingly, those with fewer or no herpes labialis outbreaks also mounted a larger immune response to *Candida* extract, even though it is unrelated to herpes—with greater PBMC proliferation to *Candida* extract and greater expression of upregulated immune genes in *Candida* extract, including IFNG, and lower expression of down‐regulated immune genes in *Candida* extract. And interestingly, SADBE application also improved the immune response to the *Candida* extract in the same ways in subjects with frequent herpes labialis outbreaks 8 weeks after SADBE application. This suggests SADBE application on the arm may also be effective to improve immune response to yeast infection anywhere in the body.

Stricker et al^37^ previously reviewed use of dinitrochlorobenzene and other contact sensitizers to treat AIDS.

## 5 SUMMARY

Those with good immune control of their HSV‐1 infection (zero herpes labialis outbreaks per year (group C); or 1 or 2 outbreaks per year (group B)) differ from those with poorer immune control (6 or more outbreaks per year, group A) in these ways:

- Greater PBMC proliferation in vitro to HSV‐1‐infected cell extracts (*P* = 0.17, not significant), HSV‐1 virus (*P* = 0.08, almost significant), *Candida* extract (*P* = 0.08, almost significant), and the three stimuli normalized and taken together (*P* < 0.01, highly significant) in group C than group A.
- Lower levels of anti‐HSV‐1 IgG in serum (*P* = 0.15, not significant, for group C vs group A and *P* = 0.04, significant, for groups B+C vs group A).
- Significantly higher expression of the IFNG gene in PBMCs in vitro stimulated with HSV‐1 virus, HSV‐1‐infected cell extracts, and Candida extract in groups B+C than group A.
- Significantly lower expression of the IL5 gene in PBMCs in vitro in unstimulated condition and stimulated with HSV‐1 virus and HSV‐1‐infected cell extracts but not with *Candida* extract in groups B+C than group A.
- Significantly higher expression of several other immune‐related genes (CCL3, CXCL5, IL1B, IL8, SPP1, FOXP3, TNF, IL12A, STAT4, CXCL10, AND STAT1) in one or more of the three stimuli in groups B+C than group A, all of which are upregulated by the same stimulus versus negative control medium; and significantly lower expression of LYZ, MX1, GATA3, and IL16 in one or more of the three stimuli in groups B+C than group A, all of which except MX1 are also downregulated by the same stimulus versus negative control medium.

Treatment of subjects with frequent outbreaks (group A) with a single topical dose of SADBE causes their serum and PBMCs to become more like those of the subjects with infrequent or zero outbreaks 56 days after the a single topical treatment with SADBE in every one of these ways than they had been on day 1 before the treatment, significantly so for each of the genes listed and highly significantly so for the normalized PBMC proliferation to the three stimuli taken together.

## ETHICAL STATEMENT

The study protocol, the investigator's brochure, and other trial‐related information were approved by an independent Institutional Review Board—Salus IRB. The study protocol was reviewed, approved, and registered at ClinicalTrials.gov under registration no. NCT03661541. The patients gave a written informed consent for the study.
